# Effects of miRNA-200b on the development of diabetic retinopathy by targeting *VEGFA* gene

**DOI:** 10.1042/BSR20160572

**Published:** 2017-03-15

**Authors:** En-Hui Li, Qin-Zhu Huang, Gao-Chun Li, Zhen-Yang Xiang, Xin Zhang

**Affiliations:** Department of Ophthalmology, Taizhou Hospital of Zhejiang Province, Taizhou 317000, P.R. China

**Keywords:** microRNA-200b, Diabetic retinopathy, Vascular endothelial growth factor A, Hepatocyte growth factor, Pigment epithelium derived factor

## Abstract

The present study explored the effect of *miR-200b* on the development of diabetic retinopathy (DR) by targeting vascular endothelial growth factor A (*VEGFA*) gene. The study populations consisted of 255 DR patients (case group) and 253 healthy people (control group), while the expressions of *miR-200b* and VEGFA mRNA were detected by quantitative real-time PCR (qRT-PCR). Bioinformatics software and dual-luciferase reporter assay were used to confirm *VEGFA* as a target gene of *miR-200b*. Also, a total of 70 Wistar male rats were selected and randomly assigned into blank, normal control (NC), *miR-200b* mimics, *miR-200b* inhibitors, *miR-200b* inhibitors + silencing vascular endothelial growth factor A (siVEGFA), and siVEGFA groups (*n*=10/group) respectively. Streptozotocin (STZ)-induced rat models of DR were successfully established. VEGFA, transforming growth factor-β1 (TGF-β1), hepatocyte growth factor (HGF), and pigment epithelium-derived factor (PEDF) were detected using qRT-PCR and Western blotting. In comparison with the control group, the case group showed lower expression of *miR-200b* but higher expression of *VEGFA* mRNA. *VEGFA* was confirmed as a target gene of *miR-200b*. Rats in the *miR-200b* mimics and siVEGFA groups exhibited higher expression of PEDF mRNA and protein but lower expressions of VEGFA, TGF-β1, HGF protein, and mRNA than the NC group. There was no remarkable difference in expressions of PEDF, VEGFA, TGF-β1, HGF protein, and mRNA between the *miR-200b* inhibitors + siVEGFA and NC groups. In conclusion, the present study demonstrated that *miR-200b* might alleviate DR development by down-regulating its target gene *VEGFA*.

## Introduction

Diabetic retinopathy (DR) is a sight-threatening chronic complication that virtually harms patients with diabetes [1]. Currently, DR affects approximately 150 million people worldwide, and the number of DR patients is expected to double by 2025 according to the World Health Organization (WHO) [2]. Patients with DR exhibited increased oxidative stress in diabetes, higher superoxide levels and damaged antioxidant defense systems in the retina and the capillary cells [3]. It is characterized by progressive alterations in the retinal microvasculature, therefore causing increased vasopermeability, non-perfusion retinal areas and pathologic intraocular proliferation of retinal vessels responding to retinal non-perfusion [1]. Destruction of impaired retina by photocoagulation has been the fundamental treatment for almost 50 years after its introduction. However, with the increasingly pandemic diabetes, new approaches are eagerly needed to understand the pathophysiology and to enhance the prevention, detection, and treatment of DR [4]. An all-round understanding of the molecular and biochemical changes in DR particularly at early stage may be conducive to new and effective therapy methods for DR prevention and amelioration [5].

miRNAs are small non-coding RNAs with ∼22 nt base pairs included and the small nucleic acids are capable of regulating gene expression, resulting in transcript degradation or translational suppression, and in the whole genome, ~30% of the genes are subjected to miRNAs regulation [6]. The *miR-200* family, including *miR-200b*, is a cluster of miRNAs, which are highly associated with epithelial–mesenchymal transition (EMT), wherein *miR-200b* was thought to be a key negative regulator of tumor metastasis, invasion, and chemo-sensitivity [7]. Dysregulation of *miR-200b* has been reported to play an essential role in the EMT and metastasis in cancers such as gastric, breast, and pancreatic carcinomas [8–10]. Vascular endothelial growth factor A (VEGFA) belongs to the cysteine knot family of growth factors, which also includes VEGFB, VEGFC, VEGFD, and placental growth factor [11]. VEGFA is a pro-angiogenic factor, which plays a role in promoting survival, migration and proliferation of endothelial cells, and enhancing vascular permeability [12]. In adults, VEGFA is indispensable for blood vessel growth, particularly in pathologies with vascular involvement and organ remodeling. For example, it involved in tumor angiogenesis, wound healing, DR, and age-related macular degeneration [13]. However, it remains unknown how VEGFA is regulated in DR. Therefore, in the present study, inclusion of DR patients and animal experiment were both performed to confirm the hypothesis that *miR-200b* may alleviate DR development by targeting *VEGFA* gene.

## Materials and methods

### Ethics statement

This research was approved by the Ethics Committee of Taizhou Hospital of Zhejiang Province and in accordance with the standards of the National Research Council. All animals were raised and treated in accordance with the Guide for the Care and Use of Laboratory Animals by National Institutes of Health of the U.S.A., and informed consent was obtained from each patient prior to study.

### Study subjects and blood sample collection

From October 2014 to July 2016, 255 patients diagnosed with DR and treated in Taizhou Hospital of Zhejiang Province were included in the case group, consisting of 134 males and 121 females (mean age 61.45 ± 11.90 years). The criteria for the diagnosis of diabetes were in accordance with the 2015 Diagnostic Criteria of Diabetes created by the American Diabetes Association (ADA) [14]. The diagnosis of patients with DR was done according to the Clinical Classification Criteria for the Diagnosis of Diabetic Retinopathy proposed in the 2002 by the Sydney International Clinical Trials Symposium [15]. All patients were to be given eye examinations (including visual acuity, intraocular pressure, fundus examination, ophthalmic B-scan ultrasonography, and slit lamp examination of the anterior segment) and a general physical checkup (including blood routine, urine routine, liver, and kidney function tests). Fasting blood glucose was controlled within 8.0 mmol/l, and 2-h post-prandial blood glucose was to be not more than 10.0 mmol/l. The exclusion criteria were as follows: no history of hepatitis, acute and chronic infection, and malignant tumor; no systemic diseases such as cardiovascular and cerebrovascular diseases, inflammatory diseases, tissue proliferative diseases, and autoimmune diseases; and no other eye infections and eye diseases. At the same time, the control group included 253 healthy people who undertook physical examination in Taizhou Hospital of Zhejiang Province, consisting of 140 males and 113 females (mean age 60.18 ± 7.68 years). Fundus photography and fundus fluorescein angiography were applied to people of the control group. Besides, their fasting blood glucose levels should have been 3.9–6.1 mmol/l and 2-h post-prandial blood glucose should have been not more than 7.0 mmol/l. The exclusion criteria were as follows: no retinopathy and other eye diseases such as age-related macular diseases and ischemic optic neuropathy; no family history of glaucoma, ocular trauma, and family history of other eye diseases. After fasting for 12–14 h, 2 ml of peripheral venous blood was extracted from all subjects. The blood was anti-coagulated with EDTA-Na2 and preserved at 4°C. After centrifugation for 15 min at the rate of 1500 rev/min and isolating the serum, the expressions of *miR-200b* and *VEGFA* mRNA were detected.

### Construction and activity detection of luciferase reporter vector

The *VEGFA* target gene fragments were inserted into wild-type VEGFA-3′-UTR-WT plasmid and mutant VEGFA-3’-UTR-MUT plasmid respectively to construct VEGFA dual-luciferase reporter gene plasmid. The targeting relationship between *miR-200b* and *VEGFA* was predicted by the biological prediction website microRNA.org and validated by dual-luciferase reporter gene assay. Then, 293T cells at the logarithmic growth phase were inoculated into 96-well plates. When the cell density reached 70%, Lipofectamine 2000 transfection was conducted to co-transfect the mixed VEGFA-3′-UTR-WT plasmid and *miR-200b* plasmid to 293T cells. The control groups (VEGFA-3′-UTR-WT+NC and VEGFA-3′-UTR-MUT + *miR-200b*) were established at the same time. After culturing for 6 h in an incubator (Thermo Fisher Scientific, San Jose, CA, U.S.A.), cells were transferred into a culture medium that contained 10% FBS to culture for another 48 h. Dual-luciferase activity was detected according to the method provided by Promega as follows: after removing cell culture fluid from 96-well plates, cells were washed softly with 100 μl of PBS. Luciferase assay reagent-I solution of 100 μl was added to each well before shaking for 15 min at room temperature, and then Glomax20/20 luminometer (Promega Corporation, Madison, WI, U.S.A.) was used for activity detection. After the measurement of firefly luciferase activity, 100 μl of luciferase assay reagent-II was added to detect the activity of renilla luciferase. Gene expressions were presented by the activity ratio of the firefly luciferase to the ranilla luciferase.

### Establishment of rat model of DR induced by streptozotocin

Seventy adult male Wistar rats (weighing 200–220 g) were purchased from the Laboratory Animal Center of China Medical University and divided into model group (*n*=60) and normal control (NC) group (*n*=10). The rats in the model group were given fasting injection with 60 mg/kg streptozotocin (STZ, dissolved in 0.01 mol/l citrate buffer solution (pH 4.4), purchased from Sigma–Aldrich Chemical Company, St. Louis MO, U.S.A.); and rats of the NC group were injected with the same dose of citrate buffer solution. Then, rats had free access to water and food under natural sunlight. After the model establishment, rats were weighed once a week and their tail vein blood were taken once every 3 days to test blood glucose. One Touch glucometer and Standard Blood Glucose Test Paper (purchased from Johnson Lifescan Inc. of U.S.A.) were used to detect the blood glucose. Rats whose glucose concentration was continually equal to or more than 16.7 mmol/l were classified into diabetic models [16] and later blood glucose measurement was conducted once a month. After 3 months of regular feeding, three randomly selected rats were given a left ventricular injection of 50 mg/ml FITC-labeled dextran (FITC–dextran, purchased from Sigma–Aldrich Chemical Company, St. Louis MO, U.S.A.). At the same time, their eyes were removed and fixed in 4% paraformaldehyde. The retinal tissues spread on glass slides were observed and photographed under a fluorescent microscope (PRIMOSTAR-FL2, Carl Zeiss MicroImaging, Inc., Thornwood, NY, U.S.A.) to identify the experimental animal model.

### Construction of *miR-200b* and *VEGFA* plasmid vectors

The mature *miR-200b* sequence (No. MI0000342) was obtained from MiRBase database to synthesize *miR-200b* mimics. *miR-200b* inhibitor was the reverse complementary sequence of mature *miR-200b* (Table 1). VEGFA silencing was in accordance with the principle of siRNA design, and homology analysis was conducted to the alternative target sequence. The NC of dsRNA was a sequence that had no homology with mammalian genome. The synthesis of oligonucleotide sequences needed to add restriction enzyme cutting sites BamHI and XhoI, which was conducted by Shanghai GenePharma Co., Ltd. Amplified fragments were recovered, linked to pM18-T vector with T4DNA ligase, and transformed into DH5α competent bacteria. Single colonies were selected for colony PCR detection. Positive colonies were selected to extract plasmid. Double enzyme digestion with *EcoR* and *KpnI* was applied to plasmid and lentiviral expression vector pMIR, which were later connected by T4 DNA ligase to form recombinant plasmids pMIR–*miR-200b* mimic, pMIR–*miR-200b* inhibitor, pMIR–siVEGFA, and pMIR-vector [17].

**Table 1:** Oligonucleotide sequences of RNA and DNA

	Sequence
*miR-200b* mimics	5′-CAUCUUACUGGGCAGCAUUGGA -3′
*miR-200b* inhibitor	5′-UCAUCAUUACCAGGCAGUAUUA-3′
siVEGFA	5′-AUGUGAAUGCAGACCAAAGAA -3′
NC	5′-AATTCTCCGAACGTGTCACGT-3′

Note: siVEGFA, silencing vascular endothelial growth factor A.

### Animal grouping

The experiment included six groups with ten rats in each group. In the blank group, the rats were given no treatment; in the NC group, the rats were injected with empty plasmid in the vitreous cavity; in the *miR-200b* mimics group, the rats were injected with *miR-200b* mimics plasmid in the vitreous cavity; in the *miR-200b* inhibitors group, the rats were injected with *miR-200b* inhibitors plasmid in the vitreous cavity; in the *miR-200b *inhibitors + si-VEGFA group, the rats were injected with *miR-200b* inhibitor and si-VEGFA plasmid in the vitreous cavity; and in the si-VEGFA group, the rats were injected with si-VEGFA plasmid in the vitreous cavity. The concentration for plasmid injection was 20 μM with 5 μl each time [18]. During detection time, the rats were killed by intraperitoneal injection with 3% pentobarbital (2 ml/kg), and their eyeballs were extracted and immersed in 10% formalin for 12 h. The crystalline lens, cornea, sclera, and choroid were removed under a dissecting microscope (Carl Zeiss MicroImaging, Inc., Thornwood, NY, U.S.A.). Free retinal tissues were obtained by removing the vitreum and the pigment epithelium on the outer layer of retina, which were preserved in liquid nitrogen for later use.

### Quantitative real-time PCR

The serum RNA was extracted by QIAamp MinElute Virus Spin Kit (Qiagen Company, Hilden, Germany) and retinal tissues of rats in each group were ground with saline. The total RNA extracted by RNA Extraction Kit (Omega Bio-tek Inc, Norcross, GA, U.S.A.) was detected for RNA purity and concentration under the ultraviolet spectrophotometer (UV-1800, Shimadzu Company, Kyoto, Japan), and observed for RNA integrity with agarose gel electrophoresis. The primers of *miR-200b*, VEGFA, transforming growth factor-β1 (TGF-β1), hepatocyte growth factor (HGF), and pigment epithelium-derived factor (PEDF) were designed with the software Primer 5.0 and synthesized by Sangon Biotech (Shanghai) Co., Ltd. (Table 2). Primescript™ RT reagent kit (Takara Biotechnology Ltd., Dalian, China) was used to reversely transcribe total RNA into cDNA, and the reverse-transcription system was 10 μl with reaction conditions as follows: 16°C for 30 min, 42°C for 30 min, and 85°C for 10 min. Quantitative real-time PCR (qRT-PCR) was conducted with SYBR^®^ premix Ex Taq TM real-time quantitative PCR Kit (Takara Biotechnology Ltd., Dalian, China) and the reaction conditions were a total of 40 cycles of pre-denaturation for 2 min at 95°C, denaturation for 5 s at 95°C, annealing for 4 s at 60°C, and extending for 30 s at 72°C. The relative expression of *miR-200b* was calculated using 2^−△△*C*^_t_ (*C*_t_, cycle threshold) with U6 sn RNA as the internal reference gene, and the mRNA expressions of VEGFA, TGF-β1, HGF, and PEDF were measured by 2^−△△*C*^_t_ with glyceraldehyde-3-phosphate dehydrogenase (*GAPDH*) as the internal reference gene.

**Table 2:** Oligonucleotide sequences

Gene	Primer sequence
*miR-200b* (rat)	R:5′-CTCCCTAAAGCCTCCCACC-3′
	R:5′-AGGGCTTTCTGCTGTTGTCC-3′
*miR-200b* (human)	F:5′-GCGGCTAATACTGCCTGGTAA-3′
	R:5′-GTGCAGGGTCCGAGGT-3′
*U6*	F:5′-CGCTTCGGCAGCACATATA-3′
	R:5′-TTCACGAATTTGCGTGTCAT-3′
*VEGFA* (rat)	F:5′-ACTTTCTGCTGTCTTGGGTG-3′
	R:5′-CTGCATGGTGATGTTGGACT-3′
*VEGFA* (human)	F:5′-CCTCCGAAACCATGAACTTT-3′
	R:5′-CCACTTCGTGATGATTCTGC-3′
*TGF-β1* (rat)	F:5′-GCCTGAGTGGCTGTCTTTTGA-3′
	R:5′-GAAGCGAAAGCCCTGTATTCC-3′
*HGF* (rat)	F:5′-GACCTTGTGAGGGAGATTAT-3′
	R:5′-ATGTGCCATCCCAAATCGTCC-3′
*PEDF* (rat)	F:5′-CCAAGTCTCTGCAGGACATGAAG-3′
	R:5′-GGTTTGCCAGTAATCTTGCTG-3′
*GAPDH*	F:5′-TGGTATCGTGGAAGGACTCA-3′
	R:5′-GCAGGGATGATGTTCTGGA-3′

### Western blotting

The retinal tissues of rats were ground with normal saline. After 15 min of centrifugation at the speed of 12000 rev/min, the supernatant was collected to conduct SDS/PAGE electrophoresis. Proteins after electrophoretic separation were transferred to the nitrocellulose filter by electrotransfer. Then 5% skimmed milk–PBS solution was closed for 1 h at room temperature and cultured at 4°C overnight with VEGF, TGF-β1, PEDF, and HGF antibody (1:500, purchased from Beijing Bosscn Company with batch number bs-0103R, bs-2202R, bs-0731R, and bs-1025P respectively). The filter was washed with PBS buffer for three times and cultured for 1 h at room temperature with HRP-cross-linked secondary antibody. The filter was then washed with PBS buffer for another three times and developed by ECL. With GAPDH as internal reference [19], the gray value ratio of target band to reference band was regarded as the relative expressions of proteins.

### ADPase histochemical method

The retinal tissues of rats in each group were obtained, cut into four valves with optic papilla as the center, rinsed for 12 h with purified water, and digested with 3% trypsin for 7 h at 37°C. Then ADPase histochemical staining was conducted as follows: the prepared retinal tissues were rinsed for 15 min five times with pre-cooling Tris-maleic acid buffer (50 mM), soaked for 15 min in Tris-maleic acid buffer (0.2 Mm, containing 1 mg/ml ADP) at 37°C, rinsed for 15 min five times with Tris-maleic acid buffer (50 mM); developed for 10 min with 1:10 sulfide; rinsed for 15 min three times with Tris-maleic acid buffer (50 mM); finally 50% glycerol was used to mount the section. Results were observed under an optical microscope (Olympus Optical Co., Ltd, Tokyo, Japan) with pictures taken by a digital camera.

### Immunohistochemistry

The retinal tissues of rats in each group were collected, embedded by paraffin, and made into sections before baking for 20 min at 68°C. After conventional xylene dewaxing and gradient alcohol dehydration, the sections were placed at room temperature for 15 min. Next, PBS was used to wash sections for 5 min two to three times before adding normal goat serum blocking solution at room temperature for 20 min. Then CD34 antibody (1:2000, purchased from Beijing Bosscn Company with batch number bs-8996R) was added for 1 h of incubation at 37°C. After PBS wash, secondary antibody was added for another hour of incubation at 37°C. Following another PBS wash, diaminobenzidine w(DAB) as used for color development and the results were observed under microscope. Then after 2 min of hematoxylin staining, sections were dehydrated, transparentized, and mounted for observation under microscope. Vascular endothelial cells were labeled with CD34 antibody [20]. A brown or yellow single endothelial cell or endothelial cell string was regarded as a blood vessel, which was used to determine the level of new blood vessel formation.

### Hematoxylin and eosin staining

The retinal tissues were fixed with Davidson’s solution for 24 h before conventional dehydration, transparentizing, wax filing, and paraffin embedding. Ten sections that were sliced continuously were 3 μm in thickness and baked for 1 h at 50°C. Routine hematoxylin and eosin (HE) staining was performed before observing retinal angiogenesis under an optical microscope. Double blind method was used to count the endothelial nuclei that broke into internal limiting membranes (only counting nuclei in close contact with the inner limiting membrane, and excluding those having no connection with the internal limiting membrane in vitreum), and average value was calculated.

### Statistical methods

The statistical analysis was conducted with SPSS 21.0. Measurement data were presented by mean ± standard deviation ($\bar{x}$ ± S.D.). Differences between groups were analyzed using *t*-test and multiple sets of data were analyzed using single factor variance analysis. Enumeration data were presented by percentage and analyzed using Chi-Square (*χ*^2^) test. *P*<0.05 was considered statistically significant.

## Results

### Baseline characteristics of subjects between the case and control groups

There were no significant differences in gender and age between the case and control groups (*P*>0.05). Patients with DR in the case group were higher than healthy people in the control group in terms of body mass index (BMI), total cholesterol, triglycerides, high-density lipoprotein, glycosylated hemoglobin, blood glucose, and blood pressure (all *P*<0.05) (Table 3).

**Table 3:** Comparisons of baseline characteristics of study subjects between the case and the control groups

Variable	Control group (*n*=253)	Case group (*n*=255)
Gender (male/female)	140/113	134/121
Age (years)	60.18 ± 7.68	61.45 ± 11.90
Disease duration (years)	–	8.21 ± 2.43
BMI (kg/m^2^)	23.83 ± 3.64	25.27 ± 3.67*
Total cholesterol (mmol/l)	4.87 ± 1.04	6.01 ± 1.04*
Triglycerides (mmol/l)	1.29 ± 0.35	2.36 ± 0.62*
High-density lipoprotein (mmol/l)	1.29 ± 0.34	1.50 ± 0.45*
Fasting blood glucose (mmol/l)	5.09 ± 1.00	9.96 ± 2.53*
Glycosylated hemoglobin (%)	5.91 ± 1.11	9.49 ± 2.04*
Systolic pressure (mmHg)	119.52 ± 14.09	134.44 ± 10.40*
Diastolic pressure (mmHg)	77.73 ± 12.37	84.14 ± 9.82*

Note: *P<0.05 compared with the control group.

### Comparisons of the expressions of *miR-200b* and *VEGFA* mRNA between the case and control groups

**Figure 1:**
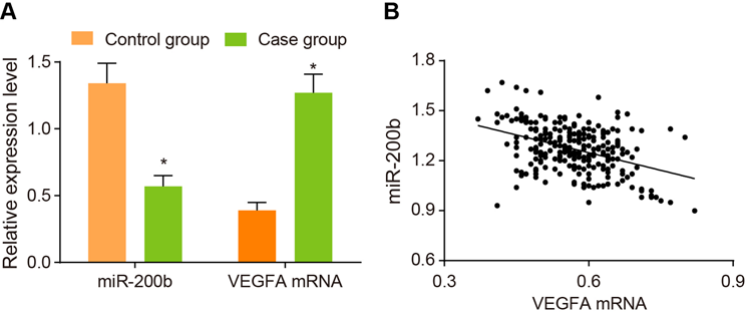
The expressions of *miR-200b* and *VEGFA* mRNA between the case and control groups and correlation analysis between *miR-200b* and VEGFA Note: (**A**) the expressions of *miR-200b* and *VEGFA* mRNA in the two groups; (**B**) correlation analysis between *miR-200b* and *VEGFA* mRNA; *P<0.05 compared with the control group.

Compared with healthy people in the control group, patients with DR in the case group significantly decreased in *miR-200b* expression and significantly increased in *VEGFA* mRNA expression (*P*<0.05) (Figure 1A). At the same time, the correlation analysis between *miR-200b* and *VGEFA* mRNA in both groups showed that *miR-200b* was negatively correlated with VGEFA (*r* = −0.4036, *P*<0.05) (Figure 1B).

### VEGFA confirmed as a target gene of *miR-200b*

**Figure 2:**
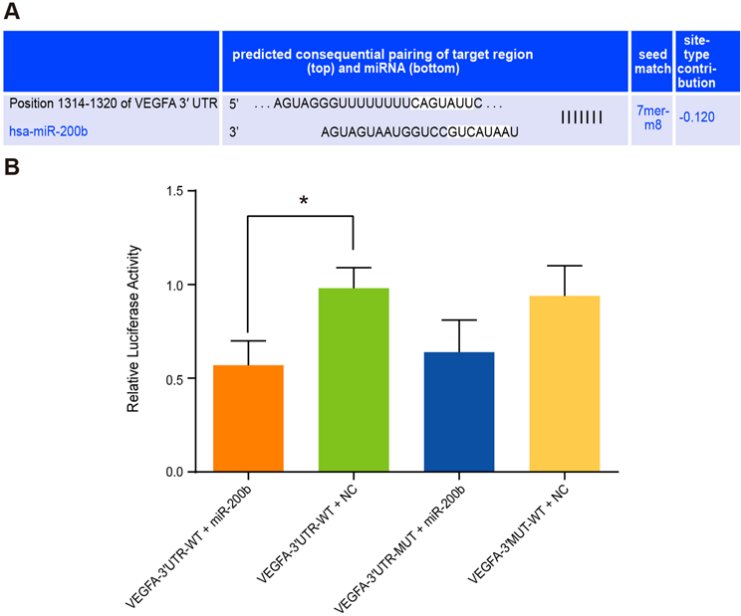
The targeting relationship between *miR-200b* and *VEGFA* gene Note: (**A**) The VEGFA 3′-UTR loci for combining *miR-200b*; (**B**) The luciferase expression was detected 48 h after 293T cells were co-transfected with VEGFA-3′-UTR-WT plasmid + *miR-200* mimics plasmid, VEGFA-3′-UTR-MUT + *miR-200b*/NC; **P<0.05 was considered statistically significant.

According to prediction of biological prediction website microRNA.org, *miR-200b* is located at the 3′-UTR intron 6 and intron 7 of *VEGFA* gene, and *miR-200b* can be partially paired with Y-UTR of *VEGFA* gene (Figure 2A). When 293T cells were co-transfected with VEGFA-3′UTR-WT plasmid and *miR-200* mimics plasmid, we found that luciferase activity ratio of firefly luciferase to ranilla luciferase (Y/H) was decreased (*P*<0.05) compared with the control group (VEGFA-3′-UTR-WT + NC) (Figure 2B). However, 293T cells co-transfected with VEGFA-3′UTR-MUT + *miR-200b* mimics plasmid showed no significant difference in Y/H with the control group (*P*>0.05). All the above findings predicted by microRNA.org indicated that *VEGFA* gene is a direct target gene of *miR-200b*.

### Successful establishment of rat models of DR induced by streptozotocin

**Figure 3:**
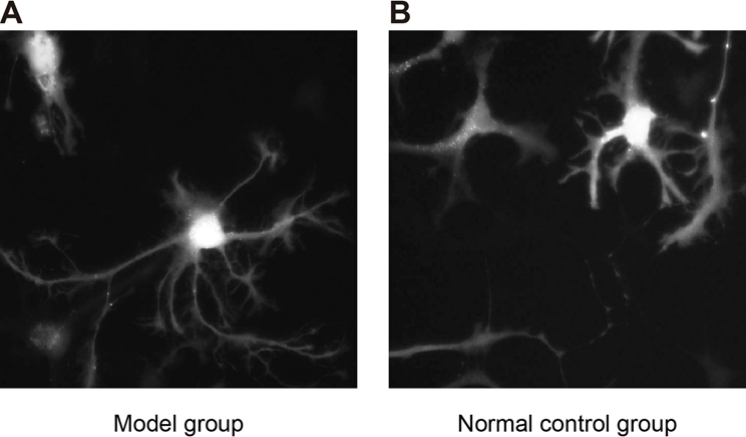
Comparisons of morphological changes of retina between the model and normal control groups (× 200) Note: (**A**) model of DR; (**B**) NC group.

The survival rate of both groups was 100%. Before model establishment, there were no significant differences in body weight and blood glucose between the model group and the NC group (all *P*>0.05). Two weeks after STZ induction, the rats in the model group manifested typical symptoms of diabetes such as obvious polydipsia, polyphagia, and polyuria. On the 8th week, apparent symptoms included lens opacification, extreme torso and head emancipation, withered hair, and abdominal swelling. On the 4th, 8th, and 12th week, the body weight of the rats in the NC group kept rising (all *P*<0.05), and their blood glucose remained stable (all *P*>0.05). Compared with the rats in the NC group, the rats in the model group significantly decreased in body weight and significantly increased in blood glucose (all *P*<0.05), and their blood glucose concentration remained at about 19 mmol/l from 4th week (Table 4). When observing tissue sections of rats after left ventricular injection with FITC–dextran, we found that model rats showed retinal capillary dilatation, interstitial edema, irregular retinal diameter, and tortuous blood vessels. However, normal rats had smooth retinal vascular branches and uniform diameter (Figure 3). The results demonstrated that the DR model was successfully established.

**Table 4:** Comparisons of body weight and blood glucose of rats in the normal control group and the model group (*X* ± S.D.)

	Time	Normal control group (*n*=10)	Model group (*n*=10)
Body weight (g)	0 week	209.76 ± 4.89	207.80 ± 8.70
	4th week	227.63 ± 14.09	204.50 ± 9.10*
	8th week	251.02 ± 21.52	191.80 ± 11.90*
	12th week	280.23 ± 25.64	171.10 ± 16.30*
Blood glucose (mmol/l)	0 week	5.40 ± 0.72	5.48 ± 0.52
	4th week	5.44 ± 0.67	18.80 ± 1.71*
	8th week	5.32 ± 0.77	21.51 ± 1.94*
	12th week	5.46 ± 0.32	25.84 ± 2.31*

Note: *P<0.05 compared with the normal control group.

### The expressions of *miR-200b* and expressions of VEGFA, TGF-β1, HGF, and PEDF mRNA of rats among six groups

**Figure 4:**
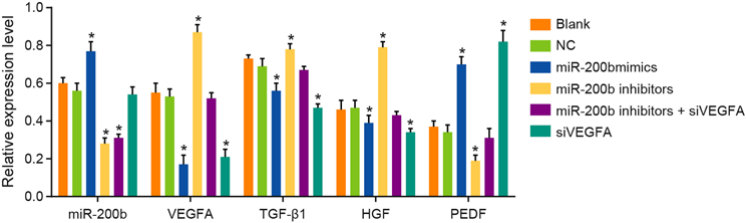
Comparisons of the expressions of *miR-200b *and VEGFA, TGF-β1, HGF, PEDF mRNA by qRT-PCR among six groups Note: *, *P* < 0.05 compared with the blank group and the NC group.

Compared with rats in the blank group and the NC group, the rats in the *miR-200b* mimics group were increased in *miR-200b* expression, while the rats in the *miR-200b* inhibitors group and the *miR-200b* inhibitors + siVEGFA group were decreased in the expression of *miR-200b* (all *P*<0.05). Compared with rats in the NC group, the rats in the *miR-200b* mimics group and the siVEGFA group were elevated in PEDF mRNA expression, and reduced in expressions of VEGFA, TGF-β1, and HGF mRNA (all *P*<0.05), but the rats in the *miR-200b* inhibitors group were decreased in PEDF mRNA expression, and increased in expressions of VEGFA, TGF-β1, and HGF mRNA (all *P*<0.05). Besides, compared with rats in the NC group, the rats in the *miR-200b* inhibitors + siVEGFA group showed no significant difference in the mRNA expressions of VEGFA, TGF-β1, HGF, and PEDF (all *P* >0.05). These results indicated that *miR-200b* over-expression and VEGFA low-expression can reduce the mRNA expression of VEGFA and retinopathy-related proteins such as TGF-β1 and HGF, and enhance the expression of PEDF mRNA. In addition, *miR-200b* was negatively correlated with VEGFA (Figure 4).

### The expressions of VEGFA, TGF-β1, HGF, and PEDF protein of rats among six groups

**Figure 5:**
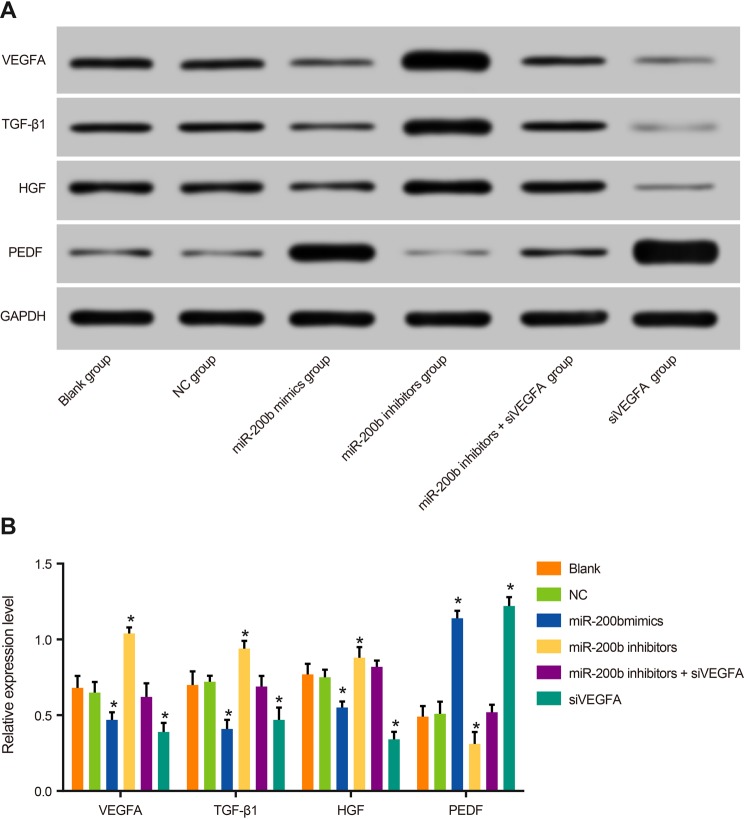
Comparisons of expressions of VEGFA and retinopathy-related proteins TGF-β1, HGF, and PEDF by Western blotting among six groups Note: *P<0.05 compared with the blank group and the NC group.

There was no significant difference in the expressions of VEGFA, TGF-β1, HGF, and PEDF proteins among the blank group, the NC group, and the *miR-200b* inhibitors + siVEGFA group (all *P*>0.05). Compared with rats in the NC group, rats in the *miR-200b* mimics group and the siVEGFA group were decreased in the expressions of VEGFA, TGF-β1, and HGF proteins, but increased in the expression of PEDF protein (all *P*<0.05). On the other hand, rats in the *miR-200b* inhibitors group were increased in the expressions of VEGFA, TGF-β1, and HGF proteins, but decreased in the expression of PEDF protein (all *P*<0.05). These results suggested that *miR-200b* over-expression and VEGFA low-expression can reduce the expression of retinopathy-related proteins such as TGF-β1 and HGF, while also enhance the expression of PEDF protein. Besides, *miR-200b* low-expression can improve the expression of retinopathy-related proteins such as TGF-β1 and HGF, and reduce the expression of PEDF protein (Figure 5).

### Morphology of retinal neovascularization of rats among six groups

**Figure 6:**
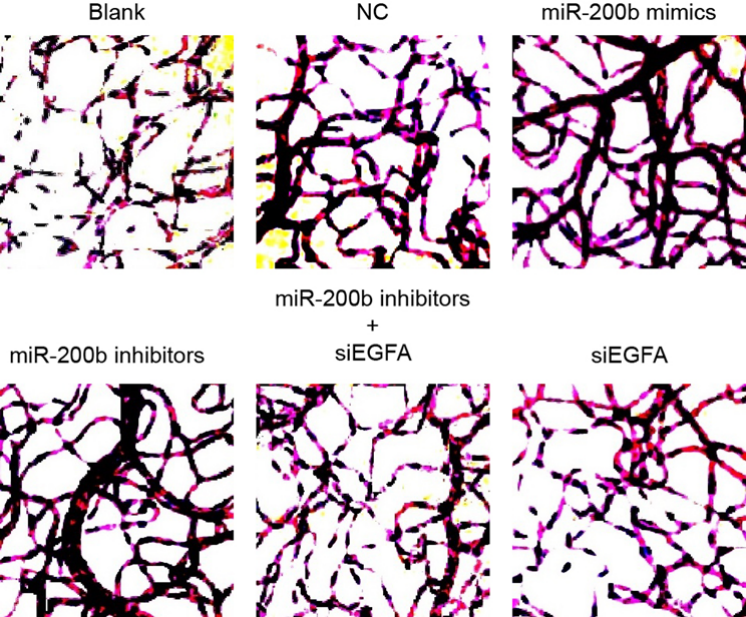
Comparisons of morphology of retinal neovascularization detected by ADPase histochemical method among six groups (× 400)

In the blank group and the NC group, rats showed dense retinal blood vessels, tortuous capillaries, irregular vessel diameter with segmental enlargement, and many visible acellular capillaries. Compared with the NC group, rats in the *miR-200b* mimics group, the siVEGFA group and the *miR-200b* inhibitors + siVEGFA group had regular blood vessel running and basically uniform capillary diameter with occasionally visible segmental expansion and few acellular capillaries. In addition, rats in the *miR-200b *inhibitors group showed tortuous blood vessel running, irregular capillary diameter and segmental enlargement but had no acellular capillaries (Figure 6).

### Retinal microvessel density of rats among six groups

**Figure 7:**
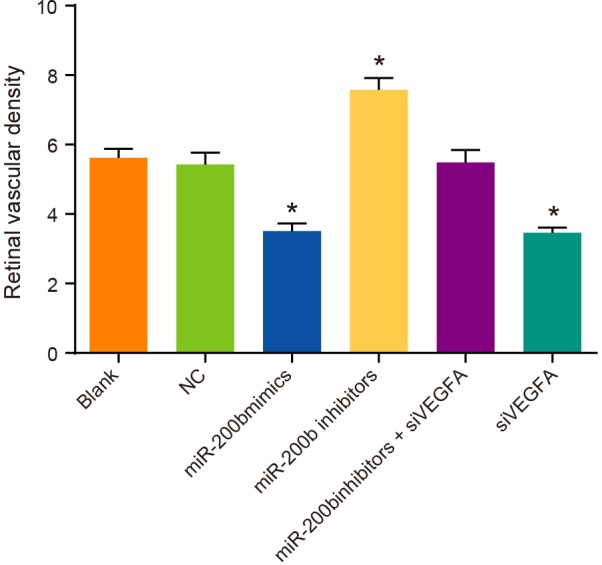
Comparisons of retinal MVD by immunohistochemistry among six groups ($\bar{x}$ ± S.D.) (× 200) Note: *P <0.05 compared with the blank group and the NC group.

There was no significant difference in retinal microvessel density (MVD) between the blank group, the NC group, and the *miR-200b* inhibitors + siVEGFA group (all *P*>0.05). Compared with rats in the NC group, rats in the *miR-200b *mimics group and the siVEGFA group had decreasing results in MVD, while the *miR-200b *inhibitors group were increased in MVD (*P*<0.05), indicating that *miR-200b* over-expression and VEGFA low-expression can reduce the growth level of MVD so as to reverse the occurrence of retinal lesions (Figure 7).

### The number of retinal vascular endothelial nuclei of rats among six groups

**Figure 8:**
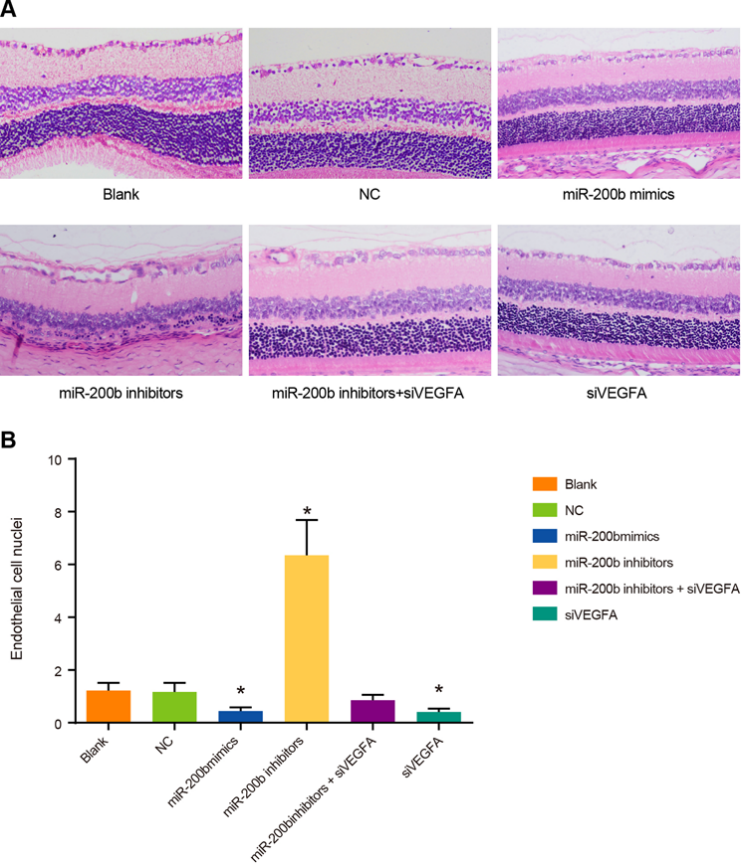
Comparisons of the number of retinal vascular endothelial nuclei by HE staining among six groups (× 200) Note: (**A**) HE staining results; (**B**) comparison of retinal vascular endothelial nuclei; *P < 0.05 compared with the blank group and the NC group.

In the blank group, the NC group and the *miR-200b* inhibitors + siVEGFA group, rats showed mild cell edema on the retinal surface, mildly disordered cell layers, a small amount of retinal neovascularization buds, and slightly irregular expanded vascular cavity. Compared with rats in the NC group, rats in the *miR-200b* mimics group and the siVEGFA group had no obvious cell edema on the retinal surface. Besides, their layers of cells were arranged in order, retinal neovascularization buds were occasionally visible, vascular cavity was regular in section, and vascular endothelial nuclei were significantly reduced (*P*<0.05). On the contrary, rats in the *miR-200b* inhibitors group showed cell edema on the retinal surface, irregularly arranged cell layers, retinal neovascularization buds, and irregularly enlarged vascular cavity section, while their vascular endothelial nuclei was significantly increased (*P*<0.05) (Figure 8). These results suggest that *miR-200b* over-expression and VEGFA low-expression can reduce the number of vascular endothelial cells and inhibit the formation of retinal neovascularization buds.

## Discussion

DR, as a common complication of diabetes, is one of the leading causes of blindness worldwide [21]. As the *VEGFA *gene has been reportedly related to the pathogenesis of DR [22], exploring the involvement of VEGFA in DR would provide a theoretical foundation for a new genetically therapeutic target against the pandemic eye disease.

In the present study, it is found that DR patients exhibited decreased expression of *miR-200b* and increased VEGFA mRNA in comparison with healthy people. miRNAs are a cluster of small non-coding RNAs capable of repressing gene expression by binding mRNA target transcripts, thus causing mRNA degradation or translational repression [23]. Being widely involved in various biologic processes, miRNAs may have an essential modulatory role in DR [24]. The presence of *miR-200b* was found in both human and rat retinas [25]. Also, our *in* *vivo* and *in* *vitro* studies demonstrating *miR-200b* expression in humans and rats suggest evolutionary conservation and it may reflect a conserved functional role in the mammalian retina. To confirm the results of the present study, McArthur et al. [24] reports that *miR-200b* is down-regulated in retinas of diabetic mice and in endothelial cells treated with high glucose. Besides, in the study, increased* VEGFA* mRNA in DR patients was also observed. The mechanisms of hyperglycemia-induced cellular damage still remain unclear. However, evidence indicates that DNA damage might be a result of high oxidative stress [26,27]. Increased oxidative stress causes the activation of the redox-sensitive transcription factors and then changes expression of many genes, including *VEGF* [28]. Several studies have demonstrated that *VEGF* gene is closely related to the severity of DR [29,30]. Whitmire et al. [31] reported that diabetic retina was companied by up-regulated VEGFA, which was in accordance with the present study. Furthermore, the study demonstrates that *miR-200b* could negatively target VEGFA. Liu et al. [32] reported that they selected mRNAs with a conserved seed sequence in their 3′-UTRs for miRNAs that were diversely expressed between tumor and normal kidney, and identified target mRNAs whose expression had an negative correlation with that of miR; they found that there was an obvious inverse correlation between the *miR-200* family and VEGF. Thus, this result provides a support to our conclusion that *VEGF* is a target gene of *miR-200b* as revealed by dual-luciferase report assay.

Additionally, in the present study, there are lower protein and mRNA expressions of VEGFA, TGF-β1, and HGF but higher mRNA expression of PEDF in *miR-200b* mimics and siVEGFA groups than in the NC group, and the *miR-200b* inhibitors group shows the opposite results. As *miR-200b* could negatively regulate VEGFA (its downstream target gene), *miR-200b *mimics group, in which rats were injected with* miR-200b* mimic plasmids, reasonably presented lower protein and mRNA expression of VEGFA. TGF-β1 is an important regulator of tissue morphogenesis and a proliferation inhibitor for most cell types [33]. As TGF-β1 protein production and mRNA expression could be blocked by VEGF via PI3K/Akt signaling [34], TGF-β1 is down-regulated accordingly with inhibited protein and mRNA expression of VEGFA. VEGFA and HGF are paracrine hormones that can regulate communication between pancreatic islet β-cells and endothelial cells [35]. VEGFA inhibition in experimental models could result in down-regulation of HGF [36], which confirms the result of the present study. However, the mechanism remains unclear. PEDF is a potent anti-angiogenic factor mediated partially by the induction of endothelial cell apoptosis [37]. PEDF can be inhibited by VEGFA through proteolytic degradation, where the lowest PEDF levels coincides with the highest VEGF-A levels and has associations with the development of retinal neovascularization in a study concerning retinal neovascularization [38]. Therefore, with down-regulated VEGFA, mRNA expression of PEDF in *miR-200b* mimics and siVEGFA groups increased. Judging by the above mechanisms, it is reasonable that 200b inhibitors group showed higher protein and mRNA expressions of VEGFA, TGF-β1, and HGF, but lower mRNA expression of PEDF.

Finally, it is observed that *miR-200b* mimics and siVEGFA groups show less circuitous blood vessels, MVD, and endothelial cell nucleus in comparison with the NC group. Expression of VEGFA could activate angiogenic response, which can further generate new and morphologically distinct blood vessels [39], while the activation of VEGFA elicits some effects on endothelial cells, such as survival, proliferation, elevated permeability, and migration [40], which is the reason why the *miR-200b* mimics and siVEGFA groups, in which VEGFA is down-regulated, exhibits decreased circuitous blood vessels, MVD, and endothelial cell nucleus. Since DR is caused by the development of abnormal new blood vessels in the retina for some extent [41], down-regulating VEGFA could be a promising strategy for the treatment of DR.

In summary, our study provided evidence that *miR-200b* might alleviate DR development by down-regulating its target gene, VEGFA. Ruiz et al. [42] have proven that the increase of trimethylation of lysine 27 on histone H3 (H3K27me3), as a special methylation, plays a regulatory role between polycomb repressive complex 2 (PRC2) and *miR-200b* on chromatin modification, and histone methyltransferase complex, PRC2 could suppress miRNAs in the development of cancers. With a high glucose environment, PRC2 could regulate the growth and proliferation of REC, improve the structure and function of vascular endothelial cells, and then slow down the deteriorating progress of DR by repressing the expression of *miR-200b *and enhancing the expression of VEGF. Consequently, it is believed that *miR-200b* may serve as a promising target in regulating VEGF-mediated abnormalities in DR. Understanding the novel mechanisms will contribute to a better understanding of DR pathogenesis and will eventually lead to formulation of specific treatment.

## Abbreviations

DR: diabetic retinopathy
EMT: epithelial–mesenchymal transition
GAPDH: glyceraldehyde-3-phosphate dehydrogenase
HE: hematoxylin and eosin
HGF: hepatocyte growth factor
MVD: microvessel density
NC: normal control
PEDF: pigment epithelium-derived factor
PRC2: polycomb repressive complex 2
qRT-PCR: quantitative real-time PCR
siVEGFA: silencing vascular endothelial growth factor A
STZ: streptozotocin
TGF-β1: transforming growth factor-β1
VEGFA: vascular endothelial growth factor A

## References

[1] Hammes H.P., Feng Y., Pfister F. and Brownlee M. (2011) Diabetic retinopathy: targeting vasoregression. Diabetes 60, 9–16 21193734 10.2337/db10-0454 PMC3012202

[2] Gupta N., Mansoor S., Sharma A., Sapkal A., Sheth J., Falatoonzadeh P. (2013) Diabetic retinopathy and VEGF. Open Ophthalmol. J. 7, 4–10 23459241 10.2174/1874364101307010004 PMC3580758

[3] Kowluru R.A., Atasi L. and Ho Y.S. (2006) Role of mitochondrial superoxide dismutase in the development of diabetic retinopathy. Invest Ophthalmol. Vis. Sci. 47, 1594–1599 16565397 10.1167/iovs.05-1276

[4] Antonetti D.A., Barber A.J., Bronson S.K., Freeman W.M., Gardner T.W., Jefferson L.S. (2006) Diabetic retinopathy: seeing beyond glucose-induced microvascular disease. Diabetes 55, 2401–2411 16936187 10.2337/db05-1635

[5] Ola M.S., Nawaz M.I., Siddiquei M.M., Al-Amro S. and Abu El-Asrar A.M. (2012) Recent advances in understanding the biochemical and molecular mechanism of diabetic retinopathy. J. Diabetes Complications 26, 56–64 22226482 10.1016/j.jdiacomp.2011.11.004

[6] Chan Y.C., Khanna S., Roy S. and Sen C.K. (2011) miR-200b targets Ets-1 and is down-regulated by hypoxia to induce angiogenic response of endothelial cells. J. Biol. Chem. 286, 2047–2056 21081489 10.1074/jbc.M110.158790 PMC3023502

[7] Feng B., Wang R. and Chen L.B. (2012) Review of miR-200b and cancer chemosensitivity. Biomed. Pharmacother. 66, 397–402 22795796 10.1016/j.biopha.2012.06.002

[8] Kurashige J., Kamohara H., Watanabe M., Hiyoshi Y., Iwatsuki M., Tanaka Y. (2012) MicroRNA-200b regulates cell proliferation, invasion, and migration by directly targeting ZEB2 in gastric carcinoma. Ann. Surg. Oncol. 19, S656–S664 22311119 10.1245/s10434-012-2217-6

[9] Gregory P.A., Bert A.G., Paterson E.L., Barry S.C., Tsykin A., Farshid G. (2008) The miR-200 family and miR-205 regulate epithelial to mesenchymal transition by targeting ZEB1 and SIP1. Nat. Cell Biol. 10, 593–601 18376396 10.1038/ncb1722

[10] Li Y., VandenBoom T.G. II, Kong D., Wang Z., Ali S., Philip P.A. (2009) Up-regulation of miR-200 and let-7 by natural agents leads to the reversal of epithelial-to-mesenchymal transition in gemcitabine-resistant pancreatic cancer cells. Cancer Res. 69, 6704–6712 19654291 10.1158/0008-5472.CAN-09-1298 PMC2727571

[11] Klein M. and Catargi B. (2007) VEGF in physiological process and thyroid disease. Ann. Endocrinol. (Paris) 68, 438–448 17991452 10.1016/j.ando.2007.09.004

[12] Ruan G.X. and Kazlauskas A. (2012) Axl is essential for VEGF-A-dependent activation of PI3K/Akt. EMBO J. 31, 1692–1703 22327215 10.1038/emboj.2012.21 PMC3321201

[13] Mackenzie F. and Ruhrberg C. (2012) Diverse roles for VEGF-A in the nervous system. Development 139, 1371–1380 22434866 10.1242/dev.072348

[14] Pinsker J.E., Shank T., Dassau E. and Kerr D. (2015, )

[15] Wilkinson C.P., Ferris F.L. III, Klein R.E., Lee P.P., Agardh C.D., Davis M. (2003) Proposed international clinical diabetic retinopathy and diabetic macular edema disease severity scales. Ophthalmology 110, 1677–1682 13129861 10.1016/S0161-6420(03)00475-5

[16] Frank R.N. (2004) Diabetic retinopathy. N. Engl. J. Med. 350, 48–58 14702427 10.1056/NEJMra021678

[17] Huang N., He Y.Q., Zhu J. and Li W.M. (2015) Construction of BAD lentivirus vector and its effect on proliferation in A549 cell lines. Sichuan Da Xue Xue Bao Yi Xue Ban 46, 363–366 26121853

[18] Kubo S. and Mitani K. (2003) A new hybrid system capable of efficient lentiviral vector production and stable gene transfer mediated by a single helper-dependent adenoviral vector. J. Virol. 77, 2964–2971 12584321 10.1128/JVI.77.5.2964-2971.2003 PMC149763

[19] Ray P.S., Jia J., Yao P., Majumder M., Hatzoglou M. and Fox P.L. (2009) A stress-responsive RNA switch regulates VEGFA expression. Nature 457, 915–919 19098893 10.1038/nature07598 PMC2858559

[20] Perez-Atayde A.R., Sallan S.E., Tedrow U., Connors S., Allred E. and Folkman J. (1997) Spectrum of tumor angiogenesis in the bone marrow of children with acute lymphoblastic leukemia. Am. J. Pathol. 150, 815–821 9060819 PMC1857903

[21] Fu Y.P., Hallman D.M., Gonzalez V.H., Klein B.E., Klein R., Hayes M.G. (2010) Identification of diabetic retinopathy genes through a Genome-Wide Association Study among Mexican-Americans from Starr County, Texas. J. Ophthalmol. 2010, 73–74 10.1155/2010/861291 PMC2939442 20871662

[22] Lu Y., Ge Y., Shi Y., Yin J. and Huang Z. (2013) Two polymorphisms (rs699947, rs2010963) in the VEGFA gene and diabetic retinopathy: an updated meta-analysis. BMC Ophthalmol. 13, 1–9 24131746 10.1186/1471-2415-13-56 PMC3852979

[23] Hon L.S. and Zhang Z. (2007) The roles of binding site arrangement and combinatorial targeting in microRNA repression of gene expression. Genome Biol. 8, R166 17697356 10.1186/gb-2007-8-8-r166 PMC2374997

[24] McArthur K., Feng B., Wu Y., Chen S. and Chakrabarti S. (2011) MicroRNA-200b regulates vascular endothelial growth factor-mediated alterations in diabetic retinopathy. Diabetes 60, 1314–1323 21357793 10.2337/db10-1557 PMC3064105

[25] Arora A., McKay G.J. and Simpson D.A. (2007) Prediction and verification of miRNA expression in human and rat retinas. Invest. Ophthalmol. Vis. Sci. 48, 3962–3967 17724173 10.1167/iovs.06-1221

[26] Adam L., Zhong M., Choi W., Qi W., Nicoloso M., Arora A. (2009) miR-200 expression regulates epithelial-to-mesenchymal transition in bladder cancer cells and reverses resistance to epidermal growth factor receptor therapy. Clin. Cancer Res. 15, 5060–5072 19671845 10.1158/1078-0432.CCR-08-2245 PMC5938624

[27] King G.L. and Loeken M.R. (2004) Hyperglycemia-induced oxidative stress in diabetic complications. Histochem. Cell Biol. 122, 333–338 15257460 10.1007/s00418-004-0678-9

[28] Feng B. and Chakrabarti S. (2012) miR-320 regulates glucose-induced gene expression in diabetes. ISRN Endocrinol. 2012, 549875 22900199 10.5402/2012/549875 PMC3415085

[29] Simo R. and Hernandez C. (2008) Intravitreous anti-VEGF for diabetic retinopathy: hopes and fears for a new therapeutic strategy. Diabetologia 51, 1574–1580 18404258 10.1007/s00125-008-0989-9

[30] Buraczynska M., Ksiazek P., Baranowicz-Gaszczyk I. and Jozwiak L. (2007) Association of the VEGF gene polymorphism with diabetic retinopathy in type 2 diabetes patients. Nephrol. Dial. Transplant. 22, 827–832 17121786 10.1093/ndt/gfl641

[31] Whitmire W., Al-Gayyar M.M., Abdelsaid M., Yousufzai B.K. and El-Remessy A.B. (2011) Alteration of growth factors and neuronal death in diabetic retinopathy: what we have learned so far. Mol. Vis. 17, 300–308 21293735 PMC3032276

[32] Liu H., Brannon A.R., Reddy A.R., Alexe G., Seiler M.W., Arreola A. (2010) Identifying mRNA targets of microRNA dysregulated in cancer: with application to clear cell Renal Cell Carcinoma. BMC Syst. Biol. 4, 51 20420713 10.1186/1752-0509-4-51 PMC2876063

[33] Ferrari G., Cook B.D., Terushkin V., Pintucci G. and Mignatti P. (2009) Transforming growth factor-beta 1 (TGF-beta1) induces angiogenesis through vascular endothelial growth factor (VEGF)-mediated apoptosis. J. Cell. Physiol. 219, 449–458 19180561 10.1002/jcp.21706 PMC2749291

[34] Lee K.S., Park S.J., Kim S.R., Min K.H., Lee K.Y., Choe Y.H. (2008) Inhibition of VEGF blocks TGF-beta1 production through a PI3K/Akt signalling pathway. Eur. Respir. J. 31, 523–531 18057050 10.1183/09031936.00125007

[35] Rozance P.J., Anderson M., Martinez M., Fahy A., Macko A.R., Kailey J. (2015) Placental insufficiency decreases pancreatic vascularity and disrupts hepatocyte growth factor signaling in the pancreatic islet endothelial cell in fetal sheep. Diabetes 64, 555–564 25249573 10.2337/db14-0462 PMC4303968

[36] Llovet J.M. (2014) Focal gains of VEGFA: candidate predictors of sorafenib response in hepatocellular carcinoma. Cancer Cell 25, 560–562 24823635 10.1016/j.ccr.2014.04.019 PMC4071286

[37] Ho T.C., Chen S.L., Yang Y.C., Liao C.L., Cheng H.C. and Tsao Y.P. (2007) PEDF induces p53-mediated apoptosis through PPAR gamma signaling in human umbilical vein endothelial cells. Cardiovasc. Res. 76, 213–223 17651710 10.1016/j.cardiores.2007.06.032

[38] Falk T., Gonzalez R.T. and Sherman S.J. (2010) The yin and yang of VEGF and PEDF: multifaceted neurotrophic factors and their potential in the treatment of Parkinson's Disease. Int. J. Mol. Sci. 11, 2875–2900 21152280 10.3390/ijms11082875 PMC2996745

[39] Nagy J.A., Feng D., Vasile E., Wong W.H., Shih S.C., Dvorak A.M. (2006) Permeability properties of tumor surrogate blood vessels induced by VEGF-A. Lab. Invest. 86, 767–780 16732297 10.1038/labinvest.3700436

[40] Covassin L.D., Villefranc J.A., Kacergis M.C., Weinstein B.M. and Lawson N.D. (2006) Distinct genetic interactions between multiple Vegf receptors are required for development of different blood vessel types in zebrafish. Proc. Natl. Acad. Sci. U.S.A. 103, 6554–6559 16617120 10.1073/pnas.0506886103 PMC1458922

[41] Raja D.S. and Vasuki S. (2015) Automatic detection of blood vessels in retinal images for diabetic retinopathy diagnosis. Comput. Math Methods Med. 2015, 419279 25810749 10.1155/2015/419279 PMC4355346

[42] Ruiz M.A., Feng B. and Chakrabarti S. (2015) Polycomb repressive complex 2 regulates mir-200b in retinal endothelial cells: Potential relevance in diabetic retinopathy. PLoS ONE 10, e0123987 25884496 10.1371/journal.pone.0123987 PMC4401764

